# The Role of Mesothelin in Gynecological Tumors and Its Significance in Targeted Therapies—A Review

**DOI:** 10.3390/cancers18111692

**Published:** 2026-05-22

**Authors:** Weronika Kawecka, Jacek R. Wilczyński, Magdalena Tyczyńska, Michał Bielak, Bogdan Obrzut, Andrzej Semczuk

**Affiliations:** 1 Department of Surgical and Oncological Gynecology, Medical University of Lodz, 92-213 Lodz, Poland; weronika.kaw98@gmail.com (W.K.); jacek.wilczynski@umed.lodz.pl (J.R.W.); 2 Doctoral School, Medical University of Lublin, 20-093 Lublin, Poland; m.tyczynska@onet.pl; 3 Child Neurology Department, Children’s Hospital University of Lublin, Medical University of Lublin, 20-093 Lublin, Poland; michal.bielak@uszd.lublin.pl; 4 Department of Obstetrics and Gynecology, Faculty of Medicine, University of Rzeszów, 35-959 Rzeszów, Poland; bogdan.obrzut@gmail.com; 5 2nd Department of Gynecological Surgery and Gynecological Oncology, Medical University of Lublin, 20-090 Lublin, Poland

**Keywords:** mesothelin, ovarian cancer, endometrial cancer, cervical cancer, target therapy

## Abstract

Mesothelin (MSLN) is a cell surface glycoprotein with limited expression in normal tissues but frequent overexpression in solid tumors, including gynecological malignancies. This review summarizes the state of the art on the biological role, diagnostic value, prognostic significance, and therapeutic potential of MSLN in ovarian, endometrial, and cervical cancers. Evidence from clinical and experimental studies indicates that MSLN contributes to tumor progression through interactions with CA125, promotion of cell adhesion and peritoneal metastasis, activation of oncogenic signaling pathways, modulation of immune responses, and development of chemoresistance. Elevated MSLN expression has been associated with advanced clinical stage of the disease, platinum resistance, and poorer survival outcomes, particularly in ovarian cancer patients, although prognostic findings remain inconsistent. Circulating soluble MSLN may serve as a minimally invasive biomarker and may improve diagnostic accuracy when combined with established markers. Therapeutic MSLN strategies—antibody-drug conjugates, CAR-T and NK cell therapies, monoclonal antibodies, immunotoxins, vaccines, and checkpoint blockade—provide promising pre-clinical and early clinical results, particularly in resistant or recurrent forms of the disease. Overall, MSLN constitutes a promising target for precision oncology in gynecological cancers, although further clinical studies are required to validate its diagnostic utility and optimize targeted therapeutic approaches.

## Simple Summary

Mesothelin is a protein that is found at high levels in several cancers and may help tumor cells grow, spread, and resist treatment. This review brings together current knowledge of mesothelin in ovarian, endometrial, and cervical cancers, focusing on its biological role, clinical value as a biomarker, and potential as a treatment target. By summarizing what is already known and where the evidence is still weak, the review aims to clarify how mesothelin may fit into future cancer research and patient care. A clearer understanding of mesothelin could help researchers develop more effective diagnostic tools, refine treatment strategies, and guide new studies in gynecologic oncology.

## 1. Introduction

Mesothelin (MSLN) is a cell surface glycoprotein that exists in two forms: the 40 kD membrane-bound protein MSLN and the soluble 31 kD protein megakaryocyte potentiating factor (MPF) [1,2,3]. It is encoded by a gene spanned at chromosome 16p13.3 [1,2,3,4,5,6]. MSLN is typically expressed at low levels in normal tissues, including the pleura, peritoneum, and pericardium, as well as in specific epithelial tissues such as the fallopian tube, endocervical mucosa, and endometrium. MSLN is frequently and highly overexpressed in various types of tumors, such as mesothelioma, ovarian, pancreatic, or lung cancer [2,7,8]. Moreover, MSLN is a specific binding protein for the cancer antigen (CA125) that mediates cell adhesion [9,10,11,12,13]. This interaction is believed to contribute to the development of peritoneal metastases [9,14,15,16,17]. Relatedly, MSLN is a promising biomarker for targeted therapies in solid tumors and can also serve as a prognostic indicator [1,18]. Despite previous findings, the significance of MSLN in gynecological tumors remains unclear, and the data are limited.

This review aims to provide a comprehensive overview of the role of MSLN in the development, prognosis, and therapeutic approaches for tumors that arise from the female genital tract. This includes an analysis of how MSLN expression influences tumor growth, its potential as a biomarker for early detection, and its implications for targeted treatment strategies in gynecological carcinomas.

Literature for this narrative review was identified through PubMed and Google Scholar searches from 1996 to 2025. Search terms combined controlled vocabulary and free text related to mesothelin, targeted therapy, and gynecological malignancies, and references from relevant review articles and included papers were also screened to identify additional studies. We included original studies and reviews relevant to mesothelin, targeted therapy, and gynecological malignancies, published in English, and selected articles based on relevance to the review question and methodological quality. Because this was a narrative review, study selection was not conducted using a formal systematic review protocol; however, the search strategy was intended to capture the most pertinent literature available.

## 2. Discussion

### 2.1. Biological Significance of Mesothelin

The *MSLN* gene, located on chromosome 16p13.3, encodes a glycoprotein precursor of two subtypes of proteins: the soluble and secreted N-terminal 31 kD MPF and the membrane-bound C-terminal 40 kD protein [1,2,4,16]. In normal tissues, MSLN is found on the pleura, pericardium, peritoneum, and in some epithelial cells of the kidney, tonsil, thymus, trachea, gallbladder, seminal vesicle, fallopian tube, uterus, and placenta [1,7,19,20,21,22]. The highest prevalence of MSLN positivity occurs in various tumor types at a relevant frequency: ovarian carcinomas, pancreatic adenocarcinoma, endometrial carcinomas, malignant mesothelioma, lung adenocarcinoma, gastric cancer, and triple-negative breast cancer [1,2,7]. In addition, MSLN expression is observed exclusively in malignant epithelioid mesotheliomas, whereas biphasic and sarcomatoid mesotheliomas generally lack mesothelin expression [1,2]. The detailed MSLN expression frequencies in gynecological malignancies (ovarian, endometrial, cervical, vaginal, and vulvar carcinomas) are presented in Table 1.

**Figure 1:**
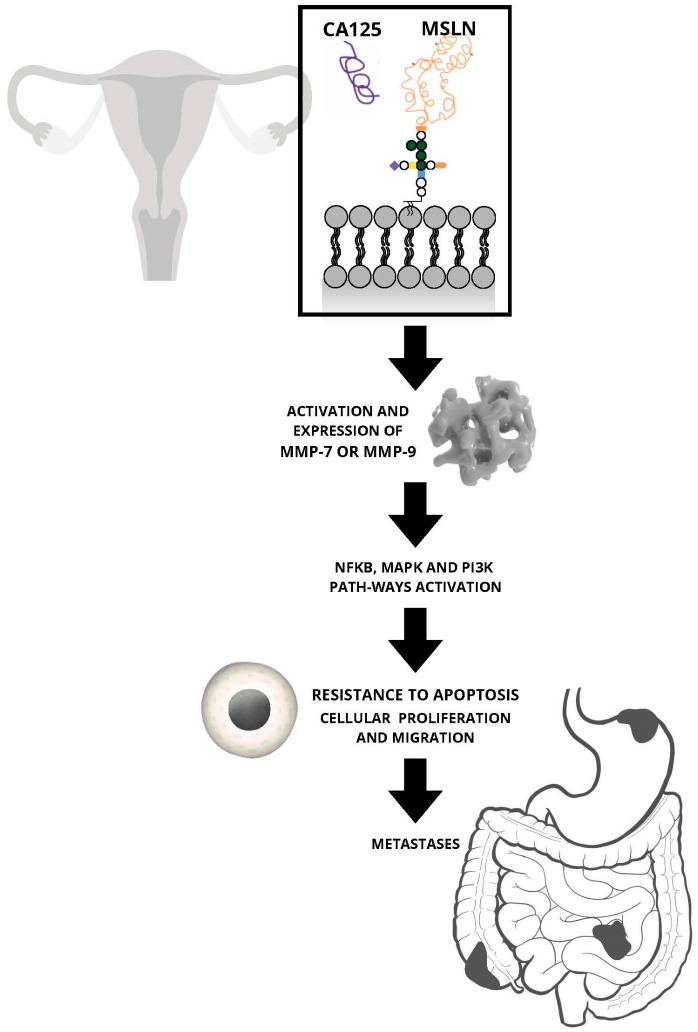
MSLN interacts with CA125 as a receptor-ligand pair to play a core role in the formation of abdominal metastases in patients affected by ovarian cancer and mesothelioma (original figure).

In normal physiology, MSLN appears to be non-essential [24]. It plays, however, a substantial role in carcinogenesis due to its interaction with mucin-16 (MUC16)/CA125. The core binding region for CA125 is located in the N-terminal fragment of mature MSLN, forming a “proline yin-yang” motif [2,16]. This primarily protein-protein interaction enables MSLN clustering on tumor cell surfaces and mediates strong cell-to-cell adhesion, which is implicated in abdominal metastasis of patients suffered from ovarian cancer and mesothelioma (Figure 1) [2,9,12,16,25]. Moreover, the overexpression of MSLN induces the activation and expression of matrix metalloproteinase 7 (MMP-7) (in ovarian and pancreatic cancers) or MMP-9 (in mesothelioma), leading to cell proliferation, migration, and the formation of metastases [18,26,27]. It could also activate the nuclear factor kappa-light-chain-enhancer of activated B cells (NFκB), mitogen-activated protein kinase (MAPK), and phosphoinositide 3-kinase (PI3K) pathways, inducing resistance to apoptosis [25,28]. It is proven that high MSLN expression correlates with immune cell infiltration in ovarian cancer: T helper cell 17 (Th17), dendritic cell (DC), and natural killer (NK) cells all have a higher degree of immune infiltration, while T helper 2 cells (Th2) and follicular helper T cells (TFH) were rarely infiltrated [29]. In addition, MSLN is positively correlated with immunosuppressive genes in ovarian cancer, including *LGALS9*, *CD276*, *TMIGD2*, *CD200*, *TNFRSF14*, and human leukocyte antigen (HLA)-related gene families, such as *HLA-DRA*, *HLA-DRB1*, *HLA-PA1*, *HLA-DMA*, and *HLA-E* [29]. Research has found that most of these immune checkpoints play a crucial role in regulating immunosuppression and antitumor activities, and that these pathways are related. These include the regulation of the immune response between the innate immune system and lymphocytes, as well as non-lymphocytes, such as the interferon gamma (IFN-γ) signaling pathway, interleukin-1-related pathways, and Th17 cell differentiation-related pathways [30,31,32]. Moreover, high MSLN expression is associated with chemoresistance, especially to platinum-based chemotherapy, and poor patients’ overall survival [8,29].

### 2.2. The Role of MSLN in Gynecological Carcinomas

#### 2.2.1. Ovarian Cancer

Ovarian cancer is the second most common cancer of female reproductive organs, right after endometrial cancer, with around 240,000–300,000 cases diagnosed and approximately 185,000 deaths annually around the world [33,34,35]. From 1990 to 2021, incidence increased from 159,096 to 298,876, and deaths increased from 100,584 to 185,609 [36]. Most cases of ovarian cancer occur in the postmenopausal period, with the peak of incidence at the age of 55–59 [33]. About 70% of ovarian cancers are detected at advanced stages (FIGO III and IV) (The International Federation of Gynecology and Obstetrics) [37]. One of the most well-known risk factors for ovarian cancer is the *BRCA1* and *BRCA2* gene mutations; 10–15% of all ovarian cancers have a genetic basis associated with these mutations [38]. About 10% of ovarian cancers are associated with Lynch syndrome [39]. Symptoms are unspecific and typically appear only in the advanced stages of the disease [40].

The diagnosis of ovarian cancer is based on medical history and specific examinations. Ovarian cancer risk is assessed using tumor marker testing, including CA125, HE4 (Human Epididymis Secretory Protein 4), and the ROMA test (Risk of Ovarian Malignancy Algorithm) [41,42,43]. Additional testing for non-epithelial ovarian cancers includes measuring the levels of human chorionic gonadotropin (beta-HCG), alpha-fetoprotein (AFP), and lactate dehydrogenase (LDH) [35]. The final confirmation of the diagnosis is based on the histopathological examination results [35].

The most widely used biomarker for ovarian cancer detection is CA125, which is secreted into the bloodstream from the coelomic and müllerian epithelia [44]. CA125 is overexpressed in more than 80% of ovarian cancer patients [45]. Furthermore, postmenopausal women with a CA125 level above 35 U/mL are considered at high risk for ovarian malignancy [46]. Additionally, the specificity of CA125 for ovarian cancer is relatively low (73–77%) [47]. The risk of ovarian cancer algorithm (ROCA) is a valuable tool for monitoring significant increases in CA125 levels and calculating ovarian cancer risk based on serial measurements [48,49]. Combining ROCA with transvaginal ultrasound enhances early detection sensitivity to at least 85% [48,49,50]. Another important biomarker is HE4, which was initially identified in the epithelium of the distal epididymis [51]. HE4 levels monitoring provides a specificity of 96% and a sensitivity of 67% in ovarian cancer detection [52]. Elevated HE4 levels are observed in over 50% of ovarian tumors that do not express CA125 [53]. Additional potential biomarkers for ovarian cancer detection include folate receptor alpha (FOLR1), CA72-4, transthyretin (TTR), CA15-3, glycodelin, kallikrein, and MSLN [43,54,55,56,57,58,59,60].

The potential use of MSLN as a biomarker for diagnosing ovarian cancer is still under research. MSLN can be found in the blood as a soluble protein (SMRP), making it a noninvasive marker [61,62]. Interestingly, MSLN can also be detected in urine, and its levels are strongly correlated with those in serum [63]. Serum MSLN levels show a moderate correlation with tumor MSLN expression [61]. Recent studies have shown that MSLN has high specificity (94%), but low sensitivity (62%) in detecting ovarian cancer. Additionally, MSLN should be used not as a single biomarker, but rather in combination with CA125 and/or HE4 to improve overall sensitivity [60]. Nevertheless, the prognostic value of MSLN expression has still not been fully determined.

In general, high MSLN expression appears to be associated with worse overall survival (OS) and progression-free survival (PFS) rates than low MSLN expression, including various histopathological types of ovarian cancer (serous, mucinous, clear cell, or endometroid) [29,61,64]. One study showed that the clinical stage IV of serous ovarian cancer, platinum resistance, higher MSLN H-score, and suboptimal surgery were associated with worse OS (67 months in the low-staining MSLN H-score vs. 27 months in the high-staining MSLN H-score) [64]. Similar results were presented in another study. Patients with epithelial ovarian carcinoma and low tumor MSLN expression demonstrated longer PFS and OS than those with high MSLN expression levels (80% vs. 40%, and 85% vs. 70%, respectively) [61]. Moreover, the high expression of MSLN was significantly correlated with a decrease in OS in different histologic subtypes at different clinical stages and at different grades of ovarian cancer [29]. On the other hand, one study has shown that positive MSLN expression is not significantly correlated with OS compared with negative MSLN (median OS 40 months vs. 34 months, respectively) [65]. Interestingly, another study found that a diffuse MSLN staining (>50% of tumor cells) in primary high-grade ovarian carcinomas correlates significantly with prolonged OS in patients who had advanced-stage disease and had received optimal debulking surgery followed by chemotherapy (60 months in patients with diffuse immunoreactivity vs. 34 months in patients with negligible or focal immunoreactivity) [66]. One study provides a more nuanced view, reporting that the presentation of MSLN on HLA-DR molecules is more significant in OS than the level of MSLN expression. Patients with a HLA-restricted presentation of high numbers of different MSLN-derived peptides on their tumors exhibited significantly prolonged PFS and OS [67].

#### 2.2.2. Endometrial Cancer

Endometrial cancer (EC) is diagnosed in approximately 417,000–470,000 women worldwide, with an increasing incidence and disease-associated mortality [68,69]. From 1990 to 2021, incidence rose from 191,291 to 473,614; deaths rose from 54,849 to 97,672. The study calls endometrial cancer the fastest-growing female-specific cancer [36]. The most common risk factors include increased age (>55 years), higher BMI, endogenous or exogenous estrogen exposure, tamoxifen use, early menarche, late menopause, and genetic predisposition (Lynch syndrome and Cowden syndrome) [70,71,72,73,74,75]. The clinical presentation involves postmenopausal bleeding, vaginal discharge, and pyometra [70,76]. The diagnosis is based on vaginal ultrasonography, endometrial biopsy, or dilation and curettage with or without hysteroscopy [77,78,79,80]. The final diagnosis is based on histopathological examination, which additionally defines the molecular subtype [69,70,81]. The histopathological and molecular endometrial cancer classification is presented in Tables S1 and S2 (Supplementary Materials) [82,83,84,85,86,87,88,89,90,91,92,93,94,95,96,97].

The management of EC includes surgical treatment as a “gold standard”, depending on the stage of the disease and other risk factors, adjuvant chemotherapy and/or radiotherapy can be used to reduce the risk of potential recurrences [98]. Total hysterectomy with bilateral salpingo-oophorectomy is the standard of care and may be performed via an open or minimally invasive approach [99,100,101,102]. Lymphadenectomy should also be considered in most cases [103].

The potential role of MSLN in endometrial cancer is still under investigation. Interestingly, MSLN is highly expressed in uterine carcinosarcomas, making it a potentially useful tool for targeted therapy [2]. Current data show no statistically significant associations between MSLN expression and clinicopathological characteristics of EC (age, FIGO stage, carcinoma component type, sarcoma component type, or receipt of chemotherapy) [23]. Interestingly, HER2-high patients are supposed to have a significantly higher MSLN expression pattern. Moreover, HER-2 status does not impact OS substantially within either the high-MSLN or low-MSLN expression groups, suggesting that MSLN may be a therapeutic target even in HER2-negative uterine tumors [23]. Additionally, MSLN expression and co-expression with CA125 might be associated with myometrial invasion, positive lymphovascular invasion, worse PFS, and OS [104]. In EC, molecular classification is important, but direct evidence linking MSLN expression to POLEmut, MMRd, NSMP, or p53abn groups remains limited in the available studies [105,106]. The potential association between MSLN expression and molecular classification of EC should be investigated.

#### 2.2.3. Cervical Cancer

Cervical cancer is estimated to have approximately 660,000 cases and 296,667 deaths globally [36,107,108]. The risk factors include HPV infection (especially HPV 16 and 18), smoking, a high number of sexual partners, and HIV infection [109,110]. Primary screening strategies in the detection of cervical cancer involve cervical cytology, HPV co-testing, and the applications of colposcopy with biopsy [111]. The most common clinical presentation is vaginal bleeding, postcoital bleeding, or intermittent spotting; however, cervical cancer may also be frequently asymptomatic [112,113]. The treatment depends on the tumor stage, histology, lymph node involvement, patient’s age, and the resources available at the treating facility, such as a multidisciplinary team or their surgical experiences [114,115].

There are a few studies that have focused on the role of MSLN in cervical cancer. It is proven that MSLN expression is increased in cervical cancer. Still, the level of expression varies by histological subtype—it seems to be the highest in non-squamous cell carcinoma. Additionally, high MSLN expression is correlated with poor prognosis [2,8]. MSLN might be an attractive therapeutic target for cervical cancer, using a combination of chemotherapy and anti-MSLN CAR-NK-92 cells [116].

To summarize, MSLN is not a single-direction prognostic marker across gynecologic cancers; the direction depends on tumor context [1]. According to tumor histotype, MSLN is enriched in some aggressive epithelial subtypes, such as cervical non-SCC and ovarian serous tumors, whereas other subtypes exhibit distinct behavior [8,66]. MSLN is best viewed as a context-dependent biomarker rather than a universally good or bad prognostic marker [1]. In particular, apparent inconsistencies in prognostic associations are considered in light of tumor histology, assay and scoring variability, differences in patient populations, and methodological limitations across studies. This approach is intended to move beyond a descriptive summary and provide a more structured interpretation of the evidence.

## 3. Targeted Therapies

MSLN seems to be one of the most promising therapeutic targets in gynecological carcinomas, due to its high, frequent, and stable expression on tumor cells and its functional role in cancer progression [117]. The most favorable candidates for MSLN-targeted therapy are patients with high MSLN expression level (>70% at 2+/3+), ≤3 prior lines of chemotherapy, and platinum-resistant or platinum-sensitive recurrent disease [118]. Currently, therapeutic options are mainly being investigated for ovarian cancer, with other types of gynecological carcinomas still lacking sufficient research and evidence. What should be emphasized is that most of the available data is associated with ovarian cancer; in the case of endometrial or cervical cancer, the data is still limited.

One of the most clinically advanced strategies for MSL-targeted therapy in ovarian cancer are antibody-drug conjugates (ADCs). They involve anetumab ravtansine, DMOT4039A, BMS-986148, and RC88 [119]. Anetumab ravtansine is a human anti-MSLN IgG1 antibody conjugated to a maytansinoid cytotoxin (DM-4) via a disulfide-containing linker. It has demonstrated the ability to kill MSLN-positive tumor cells in vivo and to have a bystander effect on nearby MSLN-negative tumor cells [120]. A favorable treatment outcome requires strong MSLN expression in tumor cells [121]. In the phase I and Ib clinical trials, the PFS totals approximately 3 months and 5 months, respectively [118,122]. The most common side effects involve nausea, corneal disorder, fatigue, gastrointestinal reactions, and anemia [118,119,122].

DMOT4039A consists of an anti-MSLN monoclonal antibody linked to monomethyl auristatin E (MMAE) [123]. A median PFS hovers around 5 months. About 20% of patients develop peripheral neuropathy [124]. BMS-986148 consists of a fully human anti-MSLN IgG1 antibody coupled to Tubulysin [125]. In a phase II trial, 9% of ovarian cancer patients achieved an objective response, and the disease control rate was 13%. The most common adverse event was hepatic transaminitis [126]. RC88 is composed of an anti-MSLN antibody coupled to a MMAE. The efficacy, safety, and pharmacokinetics of RC88 monotherapy in platinum-resistant recurrent epithelial ovarian, fallopian tube, and primary peritoneal cancer are still being evaluated in a phase II trial [127].

In CAR-T cell therapy, patients’ T cells are genetically engineered to express a synthetic receptor that targets cancer surface antigens, thereby enhancing both accuracy and effectiveness. This activates an immune response that does not rely on the major histocompatibility complex (HLA) antigen, thereby helping bypass specific tumor escape mechanisms, such as MHC-1 downregulation. Once activated, T cells secrete cytokines such as interferon-γ, perforin, and granzyme to promote cell destruction [128,129,130,131]. In one study, lentiviral-transduced CAR-T-meso cells were used in 15 patients with chemotherapy refractory solid tumors (five patients with ovarian carcinoma). A single or repeated injection of CAR-T-meso cells with or without lymphodepletion with cyclophosphamide was administered, and the best result was stable disease. The treatment was generally well tolerated [132]. Another ongoing phase I clinical trial is evaluating the use of fully human anti-MSLN M5 CAR-T cells comprising the M5 single-chain variable fragment (scFV) fused to the costimulatory CD137 and TCR zeta. In 14 patients with MSL-expressing tumors (including ovarian cancer) who received infusions with or without lymphodepletion, no objective clinical response was reported. Patients seemed to experience grade 3 cytokine release syndrome (CRS) and pulmonary adverse events [129,133]. Several studies, including individual patient reports, presented various outcomes: PFS of about 5 months or progressive disease [134,135,136]. Natural killer (NK) cells represent an alternative to CAR-T cells, particularly for the treatment of cervical cancer [137]. CAR-T cell therapies are associated with graft-versus-host disease (GvHD), neurotoxicity, and CRS. CAR-NK cells carry a much lower risk of developing GvHD or CRS due to a different cytokine profile [128,138,139,140,141,142].

Numerous clinical trials are evaluating MSLN-targeting agents, including antibody-based immunotoxins such as SS1P [62]. This immunotoxin features an anti-MLSN Fv derived from a phage display library of immunized mice, fused to a truncated Pseudomonas Exotoxin PE38 that causes cell death. Its mechanism involves three steps: binding to cell-bound MSLN, internalization through endocytosis, retrograde transport to the endoplasmic reticulum, and translocation of the PE component to the cytosol, resulting in apoptosis [117,143,144]. Two phase I trials have been conducted with different intravenous administration methods—either continuous infusion or bolus injections—in patients with mesothelioma, as well as ovarian and pancreatic cancers. Continuous infusion was generally well-tolerated and showed modest clinical activity, with some advantages over bolus dosing [145,146]. Moreover, SS1P in combination with pentostatin and cyclophosphamide induces tumor regression in patients with advanced, treatment refractory mesothelioma [62,147,148].

Additionally, a high-affinity chimeric antibody, amatuximab (MORAb-009), which targets MSLN, is currently undergoing clinical trials. Amatuximab induces antibody-dependent cellular cytotoxicity [149]. Treatment with amatuximab has been associated with elevated CA125 levels in patients, suggesting that it interferes with the MSLN:CA125 interaction [148].

Another example of targeted treatment for MSLN-positive tumors is the tumor vaccine CRS-207, which uses a live attenuated strain of Listeria monocytogenes (Lm) that produces human MSLN. It demonstrated good tolerability and induced MSLN-specific T-cell responses in a phase I safety trial. Not only did this study confirm the vaccine’s safety, but it also demonstrated that a tumor-antigen-modified Lm can elicit T-cell responses targeting tumor antigens in patients with advanced cancer. These findings suggest that further research is needed to assess the potential of the Lm vaccine as a biomarker for improved clinical outcomes [150].

The succeeding option for targeted treatment is MSLN-targeted CD47 checkpoint blockade. The CD47 immune checkpoint is significantly upregulated in several cancers to evade the immune system [151,152]. It sends a “do not eat me” signal to its co-receptor, SIRPα, which prevents phagocytosis. Targeting the CD47–SIRPα pathway is a promising approach for cancer immunotherapy [153]. In one particular study, a local inhibitory checkpoint monoclonal antibody (LicMAb) was established as a full-length anti-human MSLN-IgG1 antibody, a well-described tumor-associated antigen in epithelial ovarian cancer and pancreatic ductal adenocarcinoma. The SIRPα-αMSLN LicMAb was validated for its ability to mediate a tumor-restricted immune response, including antibody-dependent cellular cytotoxicity and phagocytosis, specifically in epithelial ovarian cancer. It was presented that cell death in epithelial ovarian cancer-derived organoids was specifically LicMAb-driven. The SIRPα-αMSLN LicMAb integrates a tumor-specific inhibition of the CD47–SIRPα pathway with targeted antitumor activity, effectively reducing on-target off-tumor toxicities. In conclusion, the multifunctional SIRPα-αMSLN LicMAb is a promising approach to treating solid tumors, especially epithelial ovarian cancer [154].

The clinical relevance of MSLN-directed therapies should be interpreted within the broader treatment landscape. Although ADCs and CAR-T approaches show encouraging activity, their current evidence base remains limited compared with established standards such as PARP inhibitors, immune checkpoint inhibitors, and other targeted agents. Accordingly, MSLN-based strategies are best viewed as emerging candidates that may complement rather than replace existing therapies, pending further validation in larger, more definitive clinical studies.

Abbreviated and simplified therapeutic approaches targeting MSLN are presented in Table 2.

## 4. Conclusions

Different aspects of MSLN role in gynecological cancers pathogenesis and management are still under investigation. High MSLN levels in tumor cells and elevated serum SMRP levels are regarded as promising diagnostic markers, especially in ovarian cancer. MSLN is also seen as a potential therapeutic target in ovarian and cervical cancers. It might also be useful as an adjuvant treatment for endometrial cancer, though more research in this area is needed. A range of therapeutic options—including ADCs, CAR-T cells, CAR-NK cells, and immunotherapy—could improve postoperative treatment. Moreover, MSLN may be considered an additional indicator of disease recurrence in post-therapeutic monitoring. Although MSLN-targeted therapies are conceptually attractive, the current clinical evidence remains limited and largely derives from early-phase studies with modest efficacy signals. Lastly, high MSLN expression is an important marker associated with poorer PFS and OS in all carcinomas. Further multicenter studies are recommended to conclusively determine the role of MSLN as a diagnostic and prognostic tool in female genital tract neoplasms.

## Figures and Tables

**Table 1:** The comparison of the histopathological type of gynecological tumors and their correlation with MSLN expression patterns [2,7,8,23].

Gynecological Tumor Subtype	MSLN-Positive Cases (%)	Strong (3+) Staining (%)	Staining Pattern
Ovary—serous carcinoma (high-grade)	94–97%	~40–90%	Predominantly apical membranous ± cytoplasmic
Ovary—serous carcinoma (low-grade)	High, similar to high-grade (exact % not always separated)	Not specified	Apical membranous
Ovary—clear cell carcinoma	~83%	Not specified	Strong membranous/apical
Ovary—endometrioid carcinoma	~77%	~33%	Membranous/apical
Ovary—mucinous carcinoma	~71%	Rare strong cases	Mostly negative or weak; rarely strong
Ovary—carcinosarcoma (OCS)	65–66%	66% high expression in the OCS cohort	Membranous
Ovary—undifferentiated carcinoma	100%	100%	Strong
Endometrium—clear cell carcinoma	~71%	Not specified	Strong membranous/apical
Endometrium—serous carcinoma	~57%	Not specified	Strong membranous
Endometrium—carcinosarcoma	~50%	33.9% high expression (≥2+, ≥30% cells)	Membranous, often in a carcinoma component
Endometrium—endometrioid carcinoma	~45%	~11%	Membranous/apical
Cervix—squamous cell carcinoma	42.4%	12.8%	Membranous/apical
Cervix—Non-SCC (adenocarcinoma, adenosquamous, etc.)	~80% adenocarcinoma	Not specified	Higher in adenocarcinoma than SCC
Vagina—squamous cell carcinoma	12%	5.3%	Membranous/apical
Vulva—squamous cell carcinoma	10.6%	2.4%	Membranous/apical

**Table 2:** Therapeutic approaches targeting MSLN [118,119,122,123,124,125,126,129,137,148,154].

Therapeutic Class/Agent	Target and Mechanism	Key Results/Efficacy in Ovarian Cancer	Toxicity & Safety Profile
ADC: anetumab ravtansine (BAY94-9343)	Anti-MSLN antibody conjugated to DM4. Induces cell death and a bystander effect.	Overall ORR: 27.7%; median PFS: 5.0 months. High-MSLN/≤3 prior therapies subgroup: ORR 42.1%, median PFS 8.5 months.	Tolerable safety profile, MTD established at 6.5 mg/kg every 3 weeks. Common side effects: nausea, corneal disorder.
ADC: DMOT4039A	Anti-MSLN antibody linked to MMAE	Among 31 high MSLN-expressing ovarian cancer patients, 3 of 10 achieved PR. Median PFS of nearly 5 months.	Microtubule-inhibitor-specific toxicity, including peripheral neuropathy (grades 1–3) in 20% of patients.
ADC: BMS-986148	Fully human anti-MSLN IgG1 coupled to Tubulysin.	Overall objective response rate (ORR) of 9% and disease control rate (DCR) of 13% in ovarian cancer patients.	Frequent hepatic adverse events (elevated AST, ALT, ALP); one patient died due to pneumonia.
CAR-T cell therapy (anti-MSLN CAR-T)	T cells genetically modified to express a chimeric antigen receptor targeting MSLN.	Modest responses; stable disease (SD) was reported in 2 out of 3 chemotherapy refractory metastatic ovarian cancer patients for 4.6 to 5.6 months. One patient had a transient tumor reduction but did not meet PR criteria.	Cytokine release syndrome (CRS): grade 3 CRS reported. Serious pulmonary adverse events due to on-target, off-tumor toxicity occurred in M5 CAR-T trials.
CAR-T cell therapy (cytokine-secreting)	Anti-MSLN CAR-T cells engineered to secrete IL-7 and CCL19	One recurrent stage III ovarian cancer patient had progressive disease by day 38.	No serious infusion or therapy-related adverse events were observed.
CAR-NK cell therapy	Natural killer (NK) cells engineered with a third-generation anti-MSLN CAR.	Demonstrated high cytotoxicity and target specificity against MSLN-expressing ovarian and cervical cancer cell lines.	Lower potential for severe CRS and neurotoxicity compared to CAR-T cells.
Monoclonal antibody (mAb): Amatuximab (MORAb-009)	Chimeric anti-MSLN IgG1 antibody that induces antibody-dependent cellular cytotoxicity (ADCC) and blocks the MSLN-CA125 adhesion interaction.	Well tolerated; demonstrated interference with the MSLN-CA125 interaction by increasing circulating CA125 levels in patients.	Well tolerated, MTD established at 200 mg/m^2^.
Engineered antibody: SIRPα–αMSLN LicMAb	Bifunctional antibody construct targeting MSLN and blocking CD47 (via low-affinity SIRPα fusion) to enhance phagocytosis.	Induced superior cytotoxicity and phagocytosis against EOC cell lines and patient-derived organoids compared to controls. Effective even in the presence of shed soluble mesothelin.	Designed to restrict CD47 blockade to tumor cells, avoiding severe on-target off-tumor toxicity seen with pan-CD47 agents.

## Data Availability

No new data were created in this review. Therefore, data sharing is not applicable to this article.

## Supplementary Materials

The following supporting information can be downloaded at: https://www.mdpi.com/article/10.3390/cancers18111692/s1, Table S1: Histopathological classification of endometrial cancer; Table S2: Molecular types of endometrial cancer.

## Abbreviations

ADCs	antibody-drug conjugates
AFP	alpha-fetoprotein
beta-HCG	beta-human chorionic gonadotropin
CA 125	cancer antigen 125
CAR	chimeric antigen receptor
CT	computer tomography
DC	dendritic cell
EC	endometrial cancer
FIGO	The International Federation of Gynecology and Obstetrics
HE4	Human Epididymis Secretory Protein 4
HLA	human leukocyte antigen
IFN-γ	interferon gamma
IL	interleukin
LDH	lactate dehydrogenase
Lm	Listeria monocytogenes
MAPK	mitogen-activated protein kinase
MMAE	monomethyl auristatin E
MMP	matrix metalloproteinase
MSLN	mesothelin
MTD	maximum tolerated dose
MPF	megakaryocyte potentiating factor
MUC16	Mucin-16
NFκB	nuclear factor kappa-light-chain-enhancer of activated B cells
NK	natural killer cell
OS	overall survival
PFS	progression-free survival
PI3K	phosphoinositide 3-kinase
PR	partial response
ROMA	risk of ovarian malignancy algorithm
SIRPα	signal regulatory protein α
TFH	follicular helper T cell
Th2	T helper cell 2
Th17	T helper cell 17
TTR	transthyretin

## References

[1] Hagerty B.L., Takabe K. (2023). Biology of mesothelin and clinical implications: A review of existing literature. World J. Oncol..

[2] Weidemann S., Gagelmann P., Gorbokon N., Lennartz M., Menz A., Luebke A.M., Kluth M., Hube-Magg C., Blessin N.C., Fraune C. (2021). Mesothelin expression in human tumors: A tissue microarray study on 12,679 tumors. Biomedicines.

[3] Hassan R., Bera T., Pastan I. (2004). Mesothelin: A new target for immunotherapy. Clin. Cancer Res..

[4] Urwin D., Lake R.A. (2000). Structure of the *Mesothelin*/*MPF* gene and characterization of its promoter. Mol. Cell Biol. Res. Commun..

[5] Yamaguchi N., Hattori K., Oh-Eda M., Kojima T., Imai N., Ochi N. (1994). A novel cytokine exhibiting megakaryocyte potentiating activity from a human pancreatic tumor cell line HPC-Y5. J. Biol. Chem..

[6] Chang K., Pastan I. (1996). Molecular cloning of mesothelin, a differentiation antigen present on mesothelium, mesotheliomas, and ovarian cancers. Proc. Natl. Acad. Sci. USA.

[7] Frierson H.F., Moskaluk C.A., Powell S.M., Zhang H., Cerilli L.A., Stoler M.H., Cathro H., Hampton G.M. (2003). Large-scale molecular and tissue microarray analysis of mesothelin expression in common human carcinomas. Hum. Pathol..

[8] Takamizawa S., Yazaki S., Kojima Y., Yoshida H., Kitadai R., Nishikawa T., Shimoi T., Sudo K., Okuma H.S., Tanioka M. (2022). High mesothelin expression is correlated with non-squamous cell histology and poor survival in cervical cancer: A retrospective study. BMC Cancer.

[9] Gubbels J.A.A., Belisle J., Onda M., Rancourt C., Migneault M., Ho M., Bera T.K., Connor J., Sathyanarayana B.K., Lee B. (2006). Mesothelin-MUC16 binding is a high affinity, N-glycan dependent interaction that facilitates peritoneal metastasis of ovarian tumors. Mol. Cancer.

[10] Scholler N., Garvik B., Hayden-Ledbetter M., Kline T., Urban N. (2007). Development of a CA125-mesothelin cell adhesion assay as a screening tool for biologics discovery. Cancer Lett..

[11] Rump A., Morikawa Y., Tanaka M., Minami S., Umesaki N., Takeuchi M., Miyajima A. (2004). Binding of ovarian cancer antigen CA125/MUC16 to mesothelin mediates cell adhesion. J. Biol. Chem..

[12] Kaneko O., Gong L., Zhang J., Hansen J.K., Hassan R., Lee B., Ho M. (2009). A binding domain on mesothelin for CA125/MUC16. J. Biol. Chem..

[13] Sasaki A., Akita K., Ito F., Mori T., Kitawaki J., Nakada H. (2015). Difference in mesothelin-binding ability of serum CA125 between patients with endometriosis and epithelial ovarian cancer. Int. J. Cancer.

[14] Avula L.R., Rudloff M., El-Behaedi S., Arons D., Albalawy R., Chen X., Zhang X., Alewine C. (2020). Mesothelin enhances tumor vascularity in newly forming pancreatic peritoneal metastases. Mol. Cancer Res..

[15] Tahara S., Nojima S., Takashima T., Okuzaki D., Morii E. (2024). Mesothelin promotes the migration of endometrioid carcinoma and is associated with the MELF pattern. Pathol. Res. Pract..

[16] Rupert P.B., Buerger M., Friend D.J., Strong R.K. (2024). Structural elucidation of the mesothelin-mucin-16/CA125 interaction. Structure.

[17] Huo Q., Xu C., Shao Y., Yu Q., Huang L., Liu Y., Bao H. (2021). Free CA125 promotes ovarian cancer cell migration and tumor metastasis by binding Mesothelin to reduce DKK1 expression and activate the SGK3/FOXO3 pathway. Int. J. Biol. Sci..

[18] Lv J., Li P. (2019). Mesothelin as a biomarker for targeted therapy. Biomark. Res..

[19] Lizio M., Abugessaisa I., Noguchi S., Kondo A., Hasegawa A., Hon C.C., de Hoon M., Severin J., Oki S., Hayashizaki Y. (2019). Update of the FANTOM web resource: Expansion to provide additional transcriptome atlases. Nucleic Acids Res..

[20] Lizio M., Harshbarger J., Shimoji H., Severin J., Kasukawa T., Sahin S., Abugessaisa I., Fukuda S., Hori F., Ishikawa-Kato S. (2015). Gateways to the FANTOM5 promoter level mammalian expression atlas. Genome Biol..

[21] Lonsdale J., Thomas J., Salvatore M., Phillips R., Lo E., Shad S., Hasz R., Walters G., Garcia F., Young Y. (2013). The Genotype-tissue expression (GTEx) project. Nat. Genet..

[22] Chang K., Pastan I., Willingham M.C. (1992). Isolation and characterization of a monoclonal antibody, K1, reactive with ovarian cancers and normal mesothelium. Int. J. Cancer.

[23] Kitadai R., Nishikawa T., Yoshida H., Mizoguchi C., Yamamoto K., Kato T., Yonemori K. (2024). Mesothelin expression in gynecologic carcinosarcoma: Clinicopathological significance and correlation with HER2 expression. J. Gynecol. Oncol..

[24] Bera T.K., Pastan I. (2000). Mesothelin is not required for normal mouse development or reproduction. Mol. Cell Biol..

[25] Tang Z., Qian M., Ho M. (2013). The role of mesothelin in tumor progression and targeted therapy. Anticancer Agents Med. Chem..

[26] Chen S.H., Hung W.C., Wang P., Paul C., Konstantopoulos K. (2013). Mesothelin binding to CA125/MUC16 promotes pancreatic cancer cell motility and invasion via MMP-7 activation. Sci. Rep..

[27] Servais E.L., Colovos C., Rodriguez L., Bograd A.J., Nitadori J., Sima C., Rusch V.W., Sadelain M., Adusumilli P.S. (2012). Mesothelin overexpression promotes mesothelioma cell invasion and MMP-9 secretion in an orthotopic mouse model and in epithelioid pleural mesothelioma patients. Clin. Cancer Res..

[28] Bharadwaj U., Marin-Muller C., Li M., Chen C., Yao Q. (2011). Mesothelin confers pancreatic cancer cell resistance to TNF-α-induced apoptosis through Akt/PI3K/NF-κB activation and IL-6/Mcl-1 overexpression. Mol. Cancer.

[29] Li Y., Tian W., Zhang H., Zhang Z., Zhao Q., Chang L., Lei N., Zhang W. (2022). MSLN correlates with immune infiltration and chemoresistance as a prognostic biomarker in ovarian cancer. Front. Oncol..

[30] Qianmei Y., Zehong S., Guang W., Hui L., Lian G. (2021). Recent advances in the role of Th17/Treg cells in tumor immunity and tumor therapy. Immunol. Res..

[31] Molgora M., Bonavita E., Ponzetta A., Riva F., Barbagallo M., Jaillon S., Popović B., Bernardini G., Magrini E., Gianni F. (2017). IL-1R8 is a checkpoint in NK cells regulating anti-tumour and anti-viral activity. Nature.

[32] Grasso C.S., Tsoi J., Onyshchenko M., Abril-Rodriguez G., Ross-Macdonald P., Wind-Rotolo M., Champhekar A., Medina E., Torrejon D.Y., Shin D.S. (2020). Conserved interferon-γ signaling drives clinical response to immune checkpoint blockade therapy in melanoma. Cancer Cell.

[33] Ali A.T., Al-Ani O., Al-Ani F. (2023). Epidemiology and risk factors for ovarian cancer. Prz. Menopauzalny.

[34] Torre L.A., Trabert B., DeSantis C.E., Miller K.D., Samimi G., Runowicz C.D., Gaudet M.M., Jemal A., Siegel R.L. (2018). Ovarian cancer statistics, 2018. CA Cancer J. Clin..

[35] Smolarz B., Biernacka K., Łukasiewicz H., Samulak D., Piekarska E., Romanowicz H., Makowska M. (2025). Ovarian cancer—Epidemiology, classification, pathogenesis, treatment, and estrogen receptors’ molecular backgrounds. Int. J. Mol. Sci..

[36] Li T., Zhang H., Lian M., He Q., Lv M., Zhai L., Zhou J., Wu K., Yi M. (2025). Global status and attributable risk factors of breast, cervical, ovarian, and uterine cancers from 1990 to 2021. J. Hematol. Oncol..

[37] Kehoe S. (2020). FIGO staging in ovarian carcinoma and histological subtypes. J. Gynecol. Oncol..

[38] Barili V., Ambrosini E., Bortesi B., Minari R., De Sensi E., Cannizzaro I.R., Taiani A., Michiara M., Sikokis A., Boggiani D. (2024). Genetic basis of breast and ovarian cancer: Approaches and lessons learnt from three decades of inherited predisposition testing. Genes.

[39] Gambini D., Ferrero S., Kuhn E. (2022). Lynch Syndrome: From carcinogenesis to prevention interventions. Cancers.

[40] Bankhead C., Collins C., Stokes-Lampard H., Rose P., Wilson S., Clements A., Mant D., Kehoe S.T., Austoker J. (2008). Identifying symptoms of ovarian cancer: A qualitative and quantitative study. BJOG.

[41] Wei S.U., Li H., Zhang B. (2016). The diagnostic value of serum HE4 and CA-125 and ROMA index in ovarian cancer. Biomed. Rep..

[42] Doubeni C.A., Doubeni A.R.B., Myers A.E. (2016). Diagnosis and management of ovarian cancer. Am. Fam. Physician.

[43] Zhang R., Siu M.K.Y., Ngan H.Y.S., Chan K.K.L. (2022). Molecular biomarkers for the early detection of ovarian cancer. Int. J. Mol. Sci..

[44] Felder M., Kapur A., Gonzalez-Bosquet J., Horibata S., Heintz J., Albrecht R., Fass L., Kaur J., Hu K., Shojaei H. (2014). MUC16 (CA125): Tumor biomarker to cancer therapy, a work in progress. Mol. Cancer.

[45] Høgdall E.V.S., Christensen L., Kjaer S.K., Blaakaer J., Kjærbye-Thygesen A., Gayther S., Jacobs I.J., Høgdall C.K. (2007). CA125 expression pattern, prognosis and correlation with serum CA125 in ovarian tumor patients: From The Danish “MALOVA” Ovarian Cancer Study. Gynecol. Oncol..

[46] Mukama T., Fortner R.T., Katzke V., Hynes L.C., Petrera A., Hauck S.M., Johnson T., Schulze M., Schiborn C., Rostgaard-Hansen A.L. (2022). Prospective evaluation of 92 serum protein biomarkers for early detection of ovarian cancer. Br. J. Cancer.

[47] Funston G., Mounce L.T.A., Price S., Rous B., Crosbie E.J., Hamilton W., Walter F.M. (2021). CA125 test result, test-to-diagnosis interval, and stage in ovarian cancer at diagnosis: A retrospective cohort study using electronic health records. Br. J. Gen. Pract..

[48] Gu Z., He Y., Zhang Y., Chen M., Song K., Huang Y., Li Q., Di W. (2018). Postprandial increase in serum CA125 as a surrogate biomarker for early diagnosis of ovarian cancer. J. Transl. Med..

[49] Gentry-Maharaj A., Blyuss O., Ryan A., Burnell M., Karpinskyj C., Gunu R., Kalsi J.K., Dawnay A., Marino I.P., Manchanda R. (2020). Multi-marker longitudinal algorithms incorporating HE4 and CA125 in ovarian cancer screening of postmenopausal women. Cancers.

[50] Lu K.H., Skates S., Hernandez M.A., Bedi D., Bevers T., Leeds L., Moore R., Granai C., Harris S., Newland W. (2013). A 2-stage ovarian cancer screening strategy using the Risk of Ovarian Cancer Algorithm (ROCA) identifies early-stage incident cancers and demonstrates high positive predictive value. Cancer.

[51] Menon U., Gentry-Maharaj A., Burnell M., Singh N., Ryan A., Karpinskyj C., Carlino G., Taylor J., Massingham S.K., Raikou M. (2021). Ovarian cancer population screening and mortality after long-term follow-up in the UK Collaborative Trial of Ovarian Cancer Screening (UKCTOCS): A randomised controlled trial. Lancet.

[52] James N.E., Chichester C., Ribeiro J.R. (2019). Beyond the biomarker: Understanding the diverse roles of human epididymis protein 4 in the pathogenesis of epithelial ovarian cancer. Front. Oncol..

[53] Li J., Dowdy S., Tipton T., Podratz K., Lu W.-G., Xie X., Jiang S.W. (2009). HE4 as a biomarker for ovarian and endometrial cancer management. Expert Rev. Mol. Diagn..

[54] Huang J., Chen J., Huang Q. (2018). Diagnostic value of HE4 in ovarian cancer: A meta-analysis. Eur. J. Obstet. Gynecol. Reprod. Biol..

[55] Leung F., Dimitromanolakis A., Kobayashi H., Diamandis E.P., Kulasingam V. (2013). Folate-receptor 1 (FOLR1) protein is elevated in the serum of ovarian cancer patients. Clin. Biochem..

[56] Zheng X., Chen S., Li L., Liu X., Liu X., Dai S., Zhang P., Lu H., Lin Z., Yu Y. (2018). Evaluation of HE4 and TTR for diagnosis of ovarian cancer: Comparison with CA-125. J. Gynecol. Obstet. Hum. Reprod..

[57] Diamandis E.P., Borgoño C.A., Scorilas A., Harbeck N., Dorn J., Schmitt M. (2004). Human kallikrein 11: An indicator of favorable prognosis in ovarian cancer patients. Clin. Biochem..

[58] Zhang X., Li M., Men X. (2020). Diagnostic value of carbohydrate antigen 72-4 combined with carbohydrate antigen 15.3 in ovarian cancer, cervical cancer and endometrial cancer. J. BUON.

[59] Scholz C., Heublein S., Lenhard M., Friese K., Mayr D., Jeschke U. (2012). Glycodelin A is a prognostic marker to predict poor outcome in advanced stage ovarian cancer patients. BMC Res. Notes.

[60] Köbel M., Madore J., Ramus S.J., Clarke B.A., Pharoah P.D.P., Deen S., Bowtell D.D., Odunsi K., Menon U., Morrison C. (2014). Evidence for a time-dependent association between FOLR1 expression and survival from ovarian carcinoma: Implications for clinical testing. An Ovarian Tumour Tissue Analysis consortium study. Br. J. Cancer.

[61] Madeira K., Dondossola E.R., de Farias B.F., Simon C.S., Alexandre M.C.M., Silva B.R., Rosa M.I. (2016). Mesothelin as a biomarker for ovarian carcinoma: A meta-analysis. An. Acad. Bras. Cienc..

[62] Hanaoka T., Hasegawa K., Kato T., Sato S., Kurosaki A., Miyara A., Nagao S., Seki H., Yasuda M., Fujiwara K. (2017). Correlation between tumor mesothelin expression and serum mesothelin in patients with epithelial ovarian carcinoma: A potential noninvasive biomarker for mesothelin-targeted therapy. Mol. Diagn. Ther..

[63] Pastan I., Hassan R. (2014). Discovery of mesothelin and exploiting it as a target for immunotherapy. Cancer Res..

[64] Schwarz F.M., Klotz D.M., Wimberger P., Kuhlmann J.D. (2024). Urinary-based detection of MSL, HE4 and CA125 as an additional dimension for predictive and prognostic modelling in ovarian cancer. Front. Oncol..

[65] Yildiz Y., Kabadayi G., Yigit S., Kucukzeybek Y., Alacacioglu A., Varol U., Taskaynatan H., Salman T., Oflazoglu U., Akyol M. (2019). High expression of mesothelin in advanced serous ovarian cancer is associated with poor prognosis. J. BUON.

[66] Magalhaes I., Fernebro J., Own S.A., Glaessgen D., Corvigno S., Remberger M., Mattsson J., Dahlstrand H. (2020). Mesothelin expression in patients with high-grade serous ovarian cancer does not predict clinical outcome but correlates with CD11c^+^ expression in tumor. Adv. Ther..

[67] Yen M.J., Hsu C.Y., Mao T.L., Wu T.C., Roden R., Wang T.L., Shih I.-M. (2006). Diffuse mesothelin expression correlates with prolonged patient survival in ovarian serous carcinoma. Clin. Cancer Res..

[68] Tegeler C.M., Scheid J., Rammensee H.G., Salih H.R., Walz J.S., Heitmann J.S., Nelde A. (2022). HLA-DR presentation of the tumor antigen MSLN associates with clinical outcome of ovarian cancer patients. Cancers.

[69] Sung H., Ferlay J., Siegel R.L., Laversanne M., Soerjomataram I., Jemal A., Bray F. (2021). Global Cancer Statistics 2020: GLOBOCAN estimates of incidence and mortality worldwide for 36 cancers in 185 Countries. CA Cancer J. Clin..

[70] Makker V., MacKay H., Ray-Coquard I., Levine D.A., Westin S.N., Aoki D., Oaknin A. (2021). Endometrial cancer. Nat. Rev. Dis. Primers.

[71] Morice P., Leary A., Creutzberg C., Abu-Rustum N., Darai E. (2016). Endometrial cancer. Lancet.

[72] Burke W.M., Orr J., Leitao M., Salom E., Gehrig P., Olawaiye A.B., Brewer M., Boruta D., Villella J., Herzog T. (2014). Endometrial cancer: A review and current management strategies: Part I. Gynecol. Oncol..

[73] Lauby-Secretan B., Scoccianti C., Loomis D., Grosse Y., Bianchini F., Straif K. (2016). International Agency for Reseach on Cancer Handbook Working Group: Body Fatness and Cancer—Viewpoint of the IARC Working Group. N. Eng. J. Med..

[74] Setiawan V.W., Yang H.P., Pike M.C., McCann S.E., Yu H., Xiang Y.B., Wolk A., Wentzensen N., Weiss N.S., Webb P.M. (2013). Type I and II endometrial cancers: Have they different risk factors?. J. Clin. Oncol..

[75] Ryan N.A.J., Glaire M.A., Blake D., Cabrera-Dandy M., Evans D.G., Crosbie E.J. (2019). The proportion of endometrial cancers associated with Lynch syndrome: A systematic review of the literature and meta-analysis. Genet. Med..

[76] Tan M.-H., Mester J.L., Ngeow J., Rybicki L.A., Orloff M.S., Eng C. (2012). Lifetime cancer risks in individuals with germline *PTEN* Mutations. Clin. Cancer Res..

[77] Clarke M.A., Long B.J., del Mar Morillo A., Arbyn M., Bakkum-Gamez J.N., Wentzensen N. (2018). Association of endometrial cancer risk with postmenopausal bleeding in women. A systematic review and meta-analysis. JAMA Intern. Med..

[78] Smith-Bindman R., Kerlikowske K., Feldstein V.A., Subak L., Scheidler J., Segal M., Brand R., Grady D. (1998). Endovaginal ultrasound to exclude endometrial cancer and other endometrial abnormalities. JAMA.

[79] Gull B., Karlsson B., Milsom I., Wikland M., Granberg S. (1996). Transvaginal sonography of the endometrium in a representative sample of postmenopausal women. Ultrasound Obstet. Gynecol..

[80] Breijer M.C., Timmermans A., van Doorn H.C., Mol B.W.J., Opmeer B.C. (2010). Diagnostic strategies for postmenopausal bleeding. Obstet. Gynecol. Int..

[81] Committee on Gynecologic Practice (2018). ACOG Committee Opinion No. 734: The role of transvaginal ultrasonography in evaluating the endometrium of women with postmenopausal bleeding. Obstet. Gynecol..

[82] Crosbie E.J., Kitson S.J., McAlpine J.N., Mukhopadhyay A., Powell M.E., Singh N. (2022). Endometrial cancer. Lancet.

[83] De Boer S.M., Wortman B.G., Bosse T., Powell M.E., Singh N., Hollema H., Wilson G., Chowdhury M.N., Mileshkin L., Pyman J. (2018). Clinical consequences of upfront pathology review in the randomised PORTEC-3 trial for high-risk endometrial cancer. Ann. Oncol..

[84] Gilks C.B., Oliva E., Soslow R.A. (2013). Poor interobserver reproducibility in the diagnosis of high-grade endometrial carcinoma. Am. J. Surg. Pathol..

[85] Brinton L.A., Felix A.S., McMeekin D.S., Creasman W.T., Sherman M.E., Mutch D., Cohn D.E., Walker J.L., Moore R.G., Downs L.S. (2013). Etiologic heterogeneity in endometrial cancer: Evidence from a Gynecologic Oncology Group trial. Gynecol. Oncol..

[86] Bokhman J.V. (1983). Two pathogenetic types of endometrial carcinoma. Gynecol. Oncol..

[87] Vermij L., Smit V., Nout R., Bosse T. (2020). Incorporation of molecular characteristics into endometrial cancer management. Histopathology.

[88] Sari A., Pollett A., Eiriksson L.R., Lumsden-Johanson B., Van de Laar E., Kazerouni H., Salehi A., Sur M., Lytwyn A., Ferguson S.E. (2019). Interobserver agreement for mismatch repair protein immunohistochemistry in endometrial and nonserous, nonmucinous ovarian carcinomas. Am. J. Surg. Pathol..

[89] Talhouk A., McConechy M.K., Leung S., Yang W., Lum A., Senz J., Boyd N., Pike J., Anglesio M., Kwon J.S. (2017). Confirmation of ProMisE: A simple, genomics-based clinical classifier for endometrial cancer. Cancer.

[90] Depreeuw J., Stelloo E., Osse E.M., Creutzberg C.L., Nout R.A., Moisse M., Garcia-Dios D.A., Dewaele M., Willekens K., Marine J.-C. (2017). Amplification of 1q32.1 refines the molecular classification of endometrial carcinoma. Clin. Cancer Res..

[91] Kurnit K.C., Kim G.N., Fellman B.M., Urbauer D.L., Mills G.B., Zhang W., Broaddus R.R. (2017). CTNNB1 (beta-catenin) mutation identifies low grade, early stage endometrial cancer patients at increased risk of recurrence. Mod. Pathol..

[92] León-Castillo A., De Boer S.M., Powell M.E., Mileshkin L.R., Mackay H.J., Leary A., Nijman H.W., Singh N., Pollock P.M., Bessette P. (2020). Molecular classification of the PORTEC-3 trial for high-risk endometrial cancer: Impact on prognosis and benefit from adjuvant therapy. J. Clin. Oncol..

[93] Xing X., Kane D.P., Bulock C.R., Moore E.A., Sharma S., Chabes A., Shcherbova P.V. (2019). A recurrent cancer-associated substitution in DNA polymerase ε produces a hyperactive enzyme. Nat. Commun..

[94] McConechy M.K., Talhouk A., Leung S., Chiu D., Yang W., Senz J., Reha-Krantz L.J., Lee C.-H., Huntsman D.G., Blake Gilks C. (2016). Endometrial carcinomas with POLE enonuclease domain mutations have a favorable prognosis. Clin. Cancer Res..

[95] Van Gool I.C., Stelloo E., Nout R.A., Nijman H.W., Edmondson R.J., Church D.N., MacKay H.J., Leary A., Powell M.E., Mileshkin L. (2016). Prognostic significance of L1CAM expression and its association with mutant p53 expression in high-risk endometrial cancer. Mod. Pathol..

[96] Bosse T., Nout R.A., Stelloo E., Dreef E., Nijman H.W., Jürgenliemk-Schulz I.M., Jobsen J.J., Creutzberg C.L., Smit V. (2014). L1 cell adhesion molecule is a strong predictor for distant recurrence and overall survival in early stage endometrial cancer: Pooled PORTEC trial results. Eur. J. Cancer.

[97] Fader A.N., Roque D.M., Siegel E., Buza N., Hui P., Abdelghany O., Chambers S., Secord A.A., Havrilesky L., O’Malley D.M. (2020). Randomized phase II trial of carboplatin–paclitaxel compared with carboplatin–paclitaxel–trastuzumab in advanced (stage III–IV) or recurrent uterine serous carcinomas that overexpress Her2/Neu (NCT01367002): Updated overall survival analysis. Clin. Cancer Res..

[98] Kuhn E., Bahadirli-Talbott A., Shih I.M. (2014). Frequent CCNE1 amplification in endometrial intraepithelial carcinoma and uterine serous carcinoma. Mod. Pathol..

[99] Brooks R.A., Fleming G.F., Lastra R.R., Lee N.K., Moroney J.W., Son C.H., Tatebe K., Veneris J.L. (2019). Current recommendations and recent progress in endometrial cancer. CA Cancer J. Clin..

[100] Walker J.L., Piedmonte M.R., Spirtos N.M., Eisenkop S.M., Schlaerth J.B., Mannel R.S., Spiegel G., Barakat R., Pearl M.L., Sharma S.K. (2009). Laparoscopy compared with laparotomy for comprehensive surgical staging of uterine cancer: Gynecologic Oncology Group Study LAP2. J. Clin. Oncol..

[101] Janda M., Gebski V., Brand A., Hogg R., Jobling T.W., Land R., Manolitsas T., McCartney A., Nascimento M., Neesham D. (2010). Quality of life after total laparoscopic hysterectomy versus total abdominal hysterectomy for stage I endometrial cancer (LACE): A randomised trial. Lancet Oncol..

[102] Galaal K., Donkers H., Bryant A., Lopes A.D. (2018). Laparoscopy versus laparotomy for the management of early stage endometrial cancer. Cochrane Database Syst. Rev..

[103] Gaia G., Holloway R.W., Santoro L., Ahmad S., Di Silverio E., Spinillo A. (2010). Robotic-assisted hysterectomy for endometrial cancer compared with traditional laparoscopic and laparotomy approaches. Obstet. Gynecol..

[104] Frost J.A., Webster K.E., Bryant A., Morrison J. (2017). Lymphadenectomy for the management of endometrial cancer. Cochrane Database Syst. Rev..

[105] Bogani G., Ray-Coquard I., Concin N., Ngoi N.Y.L., Morice P., Caruso G., Enomoto T., Takehara K., Denys H., Lorusso D. (2023). Endometrial carcinosarcoma. Int. J. Gynecol. Cancer.

[106] Huvila J., Pors J., Thompson E.F., Gilks C.B. (2021). Endometrial carcinoma: Molecular subtypes, precursors and the role of pathology in early diagnosis. J. Pathol..

[107] Kakimoto S., Miyamoto M., Einama T., Takihata Y., Matsuura H., Iwahashi H., Ishibashi H., Sakamoto T., Hada T., Suminokura J. (2021). Significance of mesothelin and CA125 expression in endometrial carcinoma: A retrospective analysis. Diagn. Pathol..

[108] Caruso G., Wagar M.K., Hsu H.C., Hoegl J., Valzacchi G.M.R., Fernandes A., Cucinella G., Aker S.S., Jayraj A.S., Mauro J. (2024). Cervical cancer: A new era. Int. J. Gynecol. Cancer.

[109] Bray F., Laversanne M., Sung H., Ferlay J., Siegel R.L., Soerjomataram I., Jemal A. (2024). Global cancer statistics 2022: GLOBOCAN estimates of incidence and mortality worldwide for 36 cancers in 185 countries. CA Cancer J. Clin..

[110] He W.Q., Li C. (2021). Recent global burden of cervical cancer incidence and mortality, predictors, and temporal trends. Gynecol. Oncol..

[111] Stelzle D., Tanaka L.F., Lee K.K., Ibrahim Khalil A., Baussano I., Shah A.S.V., McAllister D.A., Gottlieb S.L., Klug S.J., Winkler A.S. (2021). Estimates of the global burden of cervical cancer associated with HIV. Lancet Glob. Health.

[112] Viveros-Carreño D., Fernandes A., Pareja R. (2023). Updates on cervical cancer prevention. Int. J. Gynecol. Cancer.

[113] Lim A.W., Ramirez A.J., Hamilton W., Sasieni P., Patnick J., Forbes L.J. (2014). Delays in diagnosis of young females with symptomatic cervical cancer in England: An interview-based study. Br. J. Gen. Pract..

[114] Stapley S., Hamilton W. (2011). Gynaecological symptoms reported by young women: Examining the potential for earlier diagnosis of cervical cancer. Fam. Pract..

[115] Chuang L.T., Temin S., Camacho R., Dueñas-Gonzalez A., Feldman S., Gultekin M., Gupta V., Horton S., Jacob G., Kidd E.A. (2016). Management and care of women with invasive cervical cancer: American Society of Clinical Oncology resource-stratified clinical practice guideline. J. Glob. Oncol..

[116] Pujade-Lauraine E., Tan D.S.P., Leary A., Mirza M.R., Enomoto T., Takyar J., Nunes A.T., Chagüi J.D.H., Paskow M.J., Monk B.J. (2022). Comparison of global treatment guidelines for locally advanced cervical cancer to optimize best care practices: A systematic and scoping review. Gynecol. Oncol..

[117] Kutle I., Polten R., Stalp J.L., Hachenberg J., Todzey F., Hass R., Zimmermann K., von der Ohe J., von Kaisenberg C., Neubert L. (2024). Anti-Mesothelin CAR-NK cells as a novel targeted therapy against cervical cancer. Front. Immunol..

[118] Hilliard T.S. (2018). The impact of Mesothelin in the ovarian cancer tumor microenvironment. Cancers.

[119] Santin A.D., Vergote I., González-Martín A., Moore K., Oaknin A., Romero I., Diab S., Copeland L.J., Monk B.J., Coleman R.L. (2023). Safety and activity of anti-mesothelin antibody–drug conjugate anetumab ravtansine in combination with pegylated-liposomal doxorubicin in platinum-resistant ovarian cancer: Multicenter, phase Ib dose escalation and expansion study. Int. J. Gynecol. Cancer.

[120] Xu D., Chen Z.S., Peng X., Lin Z., Lu H. (2024). Research progress of antibody–drug conjugates in gynecologic cancer. Hol. Integr. Oncol..

[121] Golfier S., Kopitz C., Kahnert A., Heisler I., Schatz C.A., Stelte-Ludwig B., Mayer-Bartschmid A., Unterschemmann K., Bruder S., Linden L. (2014). Anetumab ravtansine: A novel mesothelin-targeting antibody-drug conjugate cures tumors with heterogeneous target expression favored by bystander effect. Mol. Cancer Ther..

[122] Lazzerini L., Jöhrens K., Sehouli J., Cichon G. (2020). Favorable therapeutic response after anti-Mesothelin antibody–drug conjugate treatment requires high expression of mesothelin in tumor cells. Arch. Gynecol. Obstet..

[123] Hassan R., Blumenschein G.R., Moore K.N., Santin A.D., Kindler H.L., Nemunaitis J.J., Seward S.M., Thomas A., Kim S.K., Rajagopalan P. (2020). First-in-human, multicenter, phase I dose-escalation and expansion study of anti-mesothelin antibody-drug conjugate anetumab ravtansine in advanced or metastatic solid tumors. J. Clin. Oncol..

[124] Scales S.J., Gupta N., Pacheco G., Firestein R., French D.M., Koeppen H., Rangell L., Barry-Hamilton V., Luis E., Chuhet J. (2014). An antimesothelin-monomethyl auristatin E conjugate with potent antitumor activity in ovarian, pancreatic, and mesothelioma models. Mol. Cancer Ther..

[125] Weekes C.D., Lamberts L.E., Borad M.J., Voortman J., McWilliams R.R., Diamond J.R., de Vries E.G.E., Verheul H.M., Lieu C.H., Kim G.P. (2016). Phase I study of DMOT4039A, an antibody-drug conjugate targeting mesothelin, in patients with unresectable pancreatic or platinum-resistant ovarian cancer. Mol. Cancer Ther..

[126] Clarke J., Chu S.C., Siu L.L., Machiels J.P., Markman B., Heinhuis K., Millward M., Lolkema M., Patel S.P., de Souza P. (2019). Abstract B057: BMS-986148, an anti-mesothelin antibody-drug conjugate (ADC), alone or in combination with nivolumab demonstrates clinical activity in patients with select advanced solid tumors. Mol. Cancer Ther..

[127] Rottey S., Clarke J., Aung K., Machiels J.P., Markman B., Heinhuis K.M., Millward M., Lolkema M., Patel S.P., de Souza P. (2022). Phase I/IIa trial of BMS-986148, an anti-mesothelin antibody–drug conjugate, alone or in combination with nivolumab in patients with advanced solid tumors. Clin. Cancer Res..

[128] Jiang J., Li S., Tang N., Wang L., Xin W., Li S. (2023). Preclinical safety profile of RC88-ADC a novel mesothelin-targeted antibody conjugated with monomethyl auristatin E. Drug Chem. Toxicol..

[129] June C.H., O’Connor R.S., Kawalekar O.U., Ghassemi S., Milone M.C. (2018). CAR T cell immunotherapy for human cancer. Science.

[130] Cutri-French C., Nasioudis D., George E., Tanyi J.L. (2024). CAR-T Cell Therapy in ovarian cancer: Where are we now?. Diagnostics.

[131] Maus M.V., June C.H. (2016). Making better chimeric antigen receptors for adoptive T-cell therapy. Clin. Cancer Res..

[132] June C.H., Riddell S.R., Schumacher T.N. (2015). Adoptive cellular therapy: A race to the finish line. Sci. Transl. Med..

[133] Haas A.R., Tanyi J.L., O’Hara M.H., Gladney W.L., Lacey S.F., Torigian D.A., Soulen M.C., Tian L., McGarvey M., Nelson A.M. (2019). Phase I study of lentiviral-transduced chimeric antigen receptor-modified T cells recognizing mesothelin in advanced solid cancers. Mol. Ther..

[134] Haas A.R., Golden R.J., Litzky L.A., Engels B., Zhao L., Xu F., Taraszka J.A., Ramones M., Granda B., Chang W.-J. (2023). Two cases of severe pulmonary toxicity from highly active mesothelin-directed CAR T cells. Mol. Ther..

[135] Pang N., Shi J., Qin L., Chen A., Tang Y., Yang H., Huang Y., Wu Q., Li X., He B. (2021). IL-7 and CCL19-secreting CAR-T cell therapy for tumors with positive glypican-3 or mesothelin. J. Hematol. Oncol..

[136] Fang J., Ding N., Guo X., Sun Y., Zhang Z., Xie B., Li Z., Wang H., Mao W., Lin Z. (2021). αPD-1-mesoCAR-T cells partially inhibit the growth of advanced/refractory ovarian cancer in a patient along with daily apatinib. J. Immunother. Cancer.

[137] Chen J., Hu J., Gu L., Ji F., Zhang F., Zhang M., Li J., Chen Z., Jiang L., Zhang Y. (2023). Anti-mesothelin CAR-T immunotherapy in patients with ovarian cancer. Cancer Immunol. Immunother..

[138] Neelapu S.S., Locke F.L., Bartlett N.L., Lekakis L.J., Miklos D.B., Jacobson C.A., Braunschweig I., Oluwole O.O., Siddiqi T., Lin Y. (2017). Axicabtagene ciloleucel CAR T-cell therapy in refractory large B-Cell lymphoma. N. Eng. J. Med..

[139] Sterner R.C., Sterner R.M. (2021). CAR-T cell therapy: Current limitations and potential strategies. Blood Cancer J..

[140] Klingemann H. (2014). Are natural killer cells superior CAR drivers?. Oncoimmunology.

[141] Liu E., Marin D., Banerjee P., Macapinlac H.A., Thompson P., Basar R., Kerbauy N., Overman B., Thall P., Kaplan M. (2020). Use of CAR-transduced natural killer cells in CD19-positive lymphoid tumors. N. Engl. J. Med..

[142] Lee D.W., Santomasso B.D., Locke F.L., Ghobadi A., Turtle C.J., Brudno J.N., Maus M.V., Park J.H., Mead E., Pavletic S. (2019). ASTCT consensus grading for cytokine release syndrome and neurologic toxicity associated with immune effector cells. Biol. Blood Marrow Transplant..

[143] Zhang Y., Pastan I. (2012). Modulating mesothelin shedding to improve therapy. Oncotarget.

[144] Alewine C., Hassan R., Pastan I. (2015). Advances in anticancer immunotoxin therapy. Oncologist.

[145] Kreitman R.J., Hassan R., Fitzgerald D.J., Pastan I. (2009). Phase I trial of continuous infusion anti-mesothelin recombinant immunotoxin SS1P. Clin. Cancer Res..

[146] Hassan R., Bullock S., Premkumar A., Kreitman R.J., Kindler H., Willingham M.C., Pastan I. (2007). Phase I study of SS1P, a recombinant anti-mesothelin immunotoxin given as a bolus I.V. Infusion to patients with mesothelin-expressing mesothelioma, ovarian, and pancreatic cancers. Clin. Cancer Res..

[147] Hassan R., Miller A.C., Sharon E., Thomas A., Reynolds J.C., Ling A., Kreitman R.J., Miettinen M.M., Steinberg S.M., Fowler D.H. (2013). Major cancer regressions in mesothelioma after treatment with an anti-mesothelin immunotoxin and immune suppression. Sci. Transl. Med..

[148] Hassan R., Cohen S.J., Phillips M., Pastan I., Sharon E., Kelly R.J., Schweizer C., Weil S., Laheru D. (2010). Phase I clinical trial of the chimeric anti-mesothelin monoclonal antibody MORAb-009 in patients with mesothelin-expressing cancers. Clin. Cancer Res..

[149] Hassan R., Schweizer C., Lu K.F., Schuler B., Remaley A.T., Weil S.C., Pastan I. (2010). Inhibition of mesothelin–CA-125 interaction in patients with mesothelioma by the anti-mesothelin monoclonal antibody MORAb-009: Implications for cancer therapy. Lung Cancer.

[150] Le D.T., Brockstedt D.G., Nir-Paz R., Hampl J., Mathur S., Nemunaitis J., Sterman D.H., Hassan R., Lutz E., Moyer B. (2012). A Live-attenuated listeria vaccine (ANZ-100) and a live-attenuated listeria vaccine expressing mesothelin (CRS-207) for advanced cancers: Phase I studies of safety and immune induction. Clin. Cancer Res..

[151] Willingham S.B., Volkmer J.P., Gentles A.J., Sahoo D., Dalerba P., Mitra S.S., Wang J., Contreras-Trujillo H., Martin R., Cohen J.D. (2012). The CD47-signal regulatory protein alpha (SIRPa) interaction is a therapeutic target for human solid tumors. Proc. Natl. Acad. Sci. USA.

[152] Jaiswal S., Jamieson C.H.M., Pang W.W., Park C.Y., Chao M.P., Majeti R., David Traver D., van Rooijen N., Weissman I.L. (2009). CD47 is upregulated on circulating hematopoietic stem cells and leukemia cells to avoid phagocytosis. Cell.

[153] Oldenborg P.A., Zheleznyak A., Fang Y.F., Lagenaur C.F., Gresham H.D., Lindberg F.P. (2000). Role of CD47 as a marker of self on red blood cells. Science.

[154] Reischer A., Leutbecher A., Hiller B., Perini E., White K., Hernández-Cáceres A., Schele A., Tast B., Rohrbacher L., Winter L. (2025). Targeted CD47 checkpoint blockade using a mesothelin-directed antibody construct for enhanced solid tumor-specific immunotherapy. Cancer Immunol. Immunother..

