# Supplementary material for: The Role of Mesothelin in Gynecological Tumors and Its Significance in Targeted Therapies—A Review
Source: Cancers (Basel). 2026 May 22;18(11):1692. doi: 10.3390/cancers18111692 (PMC13255823; doi:10.3390/cancers18111692)
Supplement: Supplementary file 1 [file cancers-18-01692-s001.zip › cancers-4267149-supplementary.pdf]

cancers

MDPI

Supplementary Material: The Role of Mesothelin in Gynecological Tumors and Its Significance in Targeted Therapies—A Review

Weronika Kawecka, Jacek R. Wilczyński, Magdalena Tyczyńska, Michał Bielak, Bogdan Obrzut and Andrzej Semczuk

Table S1. Histopathological classification of endometrial cancer [83–86].

Histological Type	Frequency (%)	Key Characteristics	Patients' Prognosis
Endometrioid adenocarcinoma	70–80%	Most common type; arises from atypical endometrial hyperplasia/EIN; associated with unopposed estrogen exposure; ER/PR positive	Generally favorable; 5-year OS ~85% for stage I
Serous adenocarcinoma	10–15%	Aggressive, non-estrogen-dependent; often arises from p53 signature lesions or SEIC; high nuclear grade; marked atypia	Poor; 5-year OS ~55% overall, <30% for advanced stage
Clear cell carcinoma	4–10%	Rare; often associated with endometriosis (low-grade cases); hobnail and tubulocystic architecture; usually ER/PR negative	Variable; low-grade prognosis better than high-grade; poor if p53abn subtype
Undifferentiated carcinoma	Rare (<2%)	Recently recognized; lacks all patterns of differentiation; monotonous sheet-like growth; often weakly/focally cytokeratin positive	Very poor; highly aggressive
Dedifferentiated carcinoma	Rare (<2%)	Biphasic: low-grade endometrioid EC adjacent to high-grade undifferentiated component; EMT features; frequent SWI/SNF mutations	Poor; intermediate to high mortality
Carcinosarcoma	2–5%	Biphasic malignancy with epithelial and mesenchymal (sarcomatous) components; may arise from endometrioid EC precursors or de novo p53 mutations	Very poor; 5-year OS <30%; 50–60% present at FIGO stage III–IV
Mucinous adenocarcinoma	<5%	Rare; mucin-secreting columnar epithelium; usually low-grade	Favorable (similar to low-grade endometrioid)
Mesonephric-like adenocarcinoma	<1%	Rare, recently described; tubular, ductal, papillary, and solid patterns; TP53 wild-type; MSLN likely NSMP	Aggressive; worse prognosis than NSMP endometrioid

Table S2. Molecular types of endometrial cancer [87–98].

Molecular Subtype	Frequency (%)	Key Genomic Features	Patients' Prognosis
POLEmut (POLE Ultramutated)	7–9%	Pathogenic mutation in *POLE* exonuclease domain; extremely high mutational burden (~100–500 mut/Mb); copy-number stable; high neoantigen load	**Most favorable** despite high histological grade; excellent immune response
MMRd (Mismatch Repair Deficient)	28–30%	Dysfunctional mismatch repair (MLH1, MSH2, MSH6, PMS2 mutations or MLH1 hypermethylation); 8–12 mut/Mb); high microsatellite instability (MSI-H)	**Favorable**; benefits from PD-1/PD-L1 inhibition; FDA approval of pembrolizumab
NSMP (No Specific Molecular Profile)	45–50%	Genomic stability; low copy-number alterations; POLE wild-type; MMR-proficient; TP53 wild-type; frequent *CTNNB1* mutations (associated with worse prognosis in this subtype); 1q amplifications in subset	**Favorable overall** but heterogeneous; *CTNNB1* mutations or 1q amplifications worsen outcome; low-grade tumors often have an excellent prognosis
p53abn (p53-Abnormal/Copy Number High)	15–20%	High somatic copy-number alterations; *TP53* mutations (~90%); frequent *PIK3CA*, *FBXW7*, *PPP2R1A* mutations; *CCNE1* amplification (chemoresistance); *HER2* amplification in ~20%; high genomic instability	**Poorest prognosis**; 3–5 fold higher risk of death/recurrence than p53wt; benefits from chemotherapy + radiation vs. radiation alone; emerging HER2-targeted agents

 https://doi.org/10.3390/cancers18111692
